# Identification of regions under selection and loci controlling agronomic traits in a soft red winter wheat population

**DOI:** 10.1002/tpg2.20031

**Published:** 2020-06-29

**Authors:** Rupesh Gaire, Herbert Ohm, Gina Brown‐Guedira, Mohsen Mohammadi

**Affiliations:** ^1^ Department of Agronomy Purdue University 915 West State Street West Lafayette IN 47907 USA; ^2^ Department of Crop and Soil Sciences North Carolina State University Raleigh NC 27695 USA; ^3^ US Department of Agriculture Agricultural Research Services, Southeast Area Plant Science Research Raleigh NC 27695 USA

## Abstract

Comprehensive information of a breeding population is a necessity to design promising crosses. This study was conducted to characterize a soft red winter wheat breeding population that was subject of intensive germplasm introductions and introgression from exotic germplasm. We used genome‐wide markers and phenotypic assessment to identify signatures of selection and loci controlling agronomic traits in a soft red winter wheat population. The study of linkage disequilibrium (LD) revealed that the extent of LD and its decay varied among chromosomes with chromosomes 2B and 7D showing the most extended islands of high‐LD with slow rates of decay. Four sub‐populations, two with North American origin and two with Australian and Chinese origins, were identified. Genome‐wide scans for selection signatures using F_ST_ and hapFLK identified 13 genomic regions under selection, of which five loci (*LT*, *Fr‐A2*, *Vrn‐A1*, *Vrn‐B1*, *Vrn3*) were associated with environmental adaptation and two loci were associated with disease resistance genes (*Sr36* and *Fhb1*). Genome‐wide association studies identified major loci controlling yield and yield related traits. For days to heading and plant height, major loci with effects sizes of 2.2 days and 5 cm were identified on chromosomes 7B and 6A respectively. For test weight, number of spikes per square meter, and number of kernels per square meter, large effect loci were identified on chromosomes 1A, 4B, and 5A, respectively. However, for yield alone, no major loci were detected. A combination of selection for large effect loci for yield components and genomic selection could be a promising approach for yield improvement.

**Abbreviations**
ACRE: Agronomy Center for Research and Extension
BLUE: Best linear unbiased estimate
CBF: C‐repeat binding factors
DH: Days to heading
FHB: *Fusarium* head blight
FST: Wright's Fixation index
GBS: Genotyping‐by‐sequencing
GWAS: Genome‐wide association study
LD: Linkage disequilibrium
LT: Low‐temperature tolerance
Mbp: Mega base pairs
MTA: Marker‐trait association
NK: Number of kernels per unit area
NS: Number of spikes per unit area
PCA: Principal component analysis
PH: Plant height
QTL: Quantitative trait locus
SNP: Single nucleotide polymorphism
SRW: Soft red winter
SS: Selective sweeps
TW: Test weight
YLD: Grain yield.

## 1 INTRODUCTION

Wheat occupies almost one‐fifth of world's cultivated land and is a major staple food crop for almost two‐thirds of world population. In the United States, wheat ranked third in terms of revenue and acres planted after corn and soybean (USDA‐NASS, 2019). In 2018, wheat was planted in approximately 16 million hectares producing 48 million metric tons that valued approximately $9.7 billion in the United States (USDA‐NASS, 2019). Over the last century, conventional breeding methodologies were able to exploit genetic variation in food crops that led to nearly 1% annual genetic gains in five major food crops including wheat (Ray, Mueller, West, & Foley, 2013). However, by the year 2050, global population is expected to reach 9.1 billion people, adding 1.4 billion to the current population (FAO, 2019). To meet the growing demands of food, annual cereal production should increase by 2.4% and should add one billion tons of wheat to total production every year (Ray et al., 2013). In addition, climate change brings uncertainty in crop production, anticipated to decrease wheat production over the next decade by 6% for every increase of 1°C on global temperature (Asseng et al., 2015). To address these challenges, plant breeders must leverage information from recent advancement in next‐generation sequencing technologies, bioinformatics, genomics, and phenotyping to better characterize and exploit genetic diversity for increasing genetic gains.

Existing germplasm in breeding populations are sources of native genes and desirable phenotypes. Each breeding program has several germplasm that harbor the desired adaptive genes e.g., for disease resistance or abiotic stress tolerance for breeding. When native genes are not sufficient to address environmental challenges or end‐use marketability, germplasm introduction and exotic introgression is a necessity to bring novel genes. For example, For *Fusarium* head blight (FHB) resistance, the existing native sources of resistance (Ernie, Freedom, Lyman, Overland, Jin et al., 2013) have not been sufficient to improve disease resistance. The *Fhb1* allele (Anderson et al., 2001), from the Chinese cultivar ‘Sumai 3’ has been widely introduced and incorporated in many breeding germplasms in North America (Buerstmayr, Ban, & Anderson, 2009; Jin et al., 2013).

In addition to introduction from the primary genepool, introgression of alien chromatin from wild relatives harboring useful exogenous genes has been successfully exercised to enrich the genetic bases of disease resistance. For example, barley yellow dwarf resistance gene *Bdv3* from *Thinopyrum intermedium* (Sharma et al., 1995) and the exotic *Fusarium* head blight resistance gene *Fhb7* from *Th. elongatum* (Guo et al., 2015; Shen & Ohm, 2007) were successfully transferred into the hexaploid wheat (*Triticum aestivum* L.). The introgressed alien chromatin region is not able to recombine when crossed with wild type chromatin due to lack of homologous pairing (Gill, Friebe, & White, 2011), and therefore creates an extended non‐recombinant chromatin in the chromosomal region where it is introgressed. This phenomenon will result in the extended islands of high linkage disequilibrium (Nagy et al., 2010).

**Core Ideas**

- Using GBS, ∼15000 SNP markers were developed for a soft red winter wheat population.
- Four sub‐populations were identified.
- Evidence of slow LD decay due to alien introgression was observed on chromosomes 2B and 7D.
- Selective sweeps were identified mainly in the regions previously known as environmental adaptation e.g., vernalization and low temperature tolerance.
- GWAS identified major MTAs associated with days to heading, plant height, number of spikes, and number of kernels per unit area on chromosomes 7B, 6A, 1A, and 5A respectively.

Trait‐based analyses such as bi‐parental mapping or genome‐wide association studies (GWAS) have been extensively used to identify quantitative trait loci (QTL) and genomic regions that control traits. QTL mapping is time‐ and resource‐consuming, has low mapping resolution, and does not capture all allelic diversity in germplasm (Myles et al., 2009). In contrast, GWAS identifies loci and candidate genes controlling traits in natural or breeding populations (Gupta, Kulwal, & Jaiswal, 2014). GWAS has been used in all major crops including barley (Adhikari, Steffenson, Smith, Smith, & Dill‐Macky, 2020), corn (Xiao et al., 2017), rice (Muers, 2011), soybean (Patil et al., 2017), and wheat (Gaire et al., 2019). While popular, GWAS requires phenotypic evaluation of germplasm preferentially in multiple environments and has better power to detect causative regions common (and not rare) variants within the population (Myles et al., 2009).

A complementary approach to delineate genomic regions and genes associated with adaptive responses is to identify signatures of selection or selective sweeps (SS) (Evans et al., 2014; Fariello et al., 2014; Xie et al., 2015). In plants, genome‐wide SNP markers had been used to detect SS in maize (Beissinger et al., 2014), rapeseed (Wei et al., 2017), rice (Xu et al., 2012; Xie et al., 2015), soybean (Zhou et al., 2015), tomato (Lin et al., 2014), and wheat (Cavanagh et al., 2013; Gao, Zhao, Huang, & Jia, 2017). In rice, Xie et al. (2015) evaluated breeding signatures by using ∼1500 lines, revealing that SS are enriched for adaptive traits such as semi‐dwarfism. In wheat, Cavanagh et al. (2013) used genome‐wide markers to identify signatures of domestication and post‐domestication. They estimated the extent of genetic differentiation (*F_ST_*) between spring and winter wheat and reported that *Vrn‐A1*, *Vrn‐B1*, and *Vrn‐B3* have been significantly selected. Similarly, *Ppd‐B1*, *Rht‐B1*, *Rht‐D1* and *Vrn‐A1* loci were found to be selected during domestication in Gao et al. (2017).

The population differentiation‐based method is a simple and yet powerful approach to identify SS (Vitti, Grossman, & Sabeti, 2013). When a locus has been under selection in one population but not in the other related populations, then the allele frequencies at that locus differ significantly among the populations (Holsinger & Weir, 2009). A commonly used and well‐known method in this class is the Wright's Fixation index (*F_ST_*). The *F_ST_* approach compares the variances of allele frequency within and between subpopulations (Vitti et al., 2013). Lower *F_ST_* values indicate neutral selection whereas higher *F_ST_* values are suggestive of considerable selection (Holsinger & Weir, 2009). In other words, higher values of *F_ST_* mean that the variations among subpopulations outyields the variations within subpopulations.

While commonly used, *F_ST_* method does not account for heterogeneity of sub‐population sizes, shared ancestry, and hierarchical structure of subpopulations (Bonhomme et al., 2010; Fariello, Boitard, Naya, SanCristobal, & Servin, 2013; Holsinger & Weir, 2009). That is, regions may be identified that are inherent to these underlying factors rather than differentiation, which results in the identification of false positive regions. Bonhomme et al. (2010) suggested to account for hierarchical structure and heterogeneity of effective population size. They modified the *F_ST_* approach to FLK test where allele frequencies are first rescaled by using a population kinship matrix based on the Reynolds’ distance (Reynolds, Weir, & Cockerham, 1983). Later, Fariello et al. (2013) modified FLK test such that instead of SNP frequencies, haplotype frequencies are used in the estimating *F_ST_*. Therefore, the *F_ST_* statistics which is after consideration of hierarchical structure and heterogeneity of effective population size, and uses haplotype frequency rather than SNP frequency, is called hapFLK. The hapFLK approach has been commonly used in breeding of animals such as sheep (Fariello et al., 2014), goat (Brito et al., 2017), cattle (Yurchenko et al., 2018), and chickens (Brito et al., 2017; Gholami et al., 2015); Gholami et al., 2015). In sheep, Fariello et al., 2014 identified SS that were selected for morphology, color, and adaptation. In goat, Brito et al. (2017) identified SS for fertility, reproduction and adult body mass. This approach can be extended to evaluate selection history of crop plants when phenotyping large experimental populations without the need to phenotype the population in large experimental settings.

In this study, we examine genetic diversity in a US soft red winter (SRW) wheat breeding population developed at small grain breeding program at Purdue University, which released cultivars as early as 1920s. During the 1970s, cultivars release from this program covered more than 80% of SRW wheat acreages in the United States (Ohm & McFee, 2012). Given such breeding history which involved germplasm introduction and alien introgression for various biotic stress resistance genes, the objectives of this study were to characterize the genotypic and phenotypic diversity of the germplasm, as related to environmental adaptation and agronomic traits.

## 2 MATERIALS AND METHODS

### 2.1 Genotyping

We genotyped 436 SRW wheat germplasm from Purdue's small grain breeding population. The materials were developed from 123 crosses conducted from 1988 to 2010. Leaf samples from single plants grown in the greenhouse were used for genomic DNA extraction and preparing reduced representation libraries following *Pst1‐Msp1* digestion (Poland et al. 2012). The libraries were 144‐plex sequenced on three lanes of Illumina Hi‐Seq 2500. The NGS data was processed using TASSEL 5 (Bradbury et al., 2007) GBS v2 Pipeline. The reads were aligned to IWGSC RefSeq assembly v1.0 (Appels et al., 2018). We removed single nucleotide polymorphisms (SNPs) that had heterozygosity greater than 10%, more than 20% missing data, and those with less than 5% of minor allele frequency. Then, the remaining missing genotypic data was imputed using LDKNNimp method (Money et al., 2015) implemented in TASSELv 5.2 (Bradbury et al., 2007).

The population was genotyped for major genes at USDA‐ARS Eastern Regional Small Grains Genotyping Lab, with the primer sequences indicated by Ward et al. (2019). Briefly, we genotyped the population for the presence of 1RS:1BL translocations from rye (*Secale cereale* L.) and stem rust resistance *Sr36*. In addition, allelic variations of photoperiod loci located on the group 2 homoeologous chromosomes *Ppd‐1*, reduced height locus *Rht‐B1* and *Rht‐D1* located on 4B and 4D respectively, and vernalization *Vrn1* loci for spring and winter allele located on the group 5 homoeologous chromosomes were assessed. We also genotyped for a SNP located on the 4^th^ exon of *vrn‐A1* that discriminates the *vrn‐A1a* short vernalization requiring phenotype against the long vernalization requiring, and *vrn‐A1b* (Chen, Carver, Wang, Zhang, & Yan, 2009).

### 2.2 Population structure and the extent of linkage disequilibrium

Population structure was analyzed using admixture model‐based clustering method implemented in STRUCTURE v2.3 (Pritchard, Wen, & Falush, 2003). A total of 100,000 burn‐in periods followed by 100,000 Markov Chain‐Monte Carlo iterations for K values from 1 to 10, with five independent runs for each K was used. Markers with linkage disequilibrium (LD) *r^2^* > 0.5 within a 50 SNPs window size were trimmed using ‘–indep‐pairwise’ function in PLINK 1.9 (Chang et al., 2015). The optimal number of subpopulations were determined from delta K (Δk) model as proposed by Evanno, Regnaut, and Goudet (2005). The relationship between the subpopulations identified by STRUCTURE was illustrated by the neighbor‐joining algorithm and a genomic population trees which uses pair‐wise population Reynold's distance (Reynolds et al., 1983) estimated from all markers. Estimation of Reynolds distance and drawing was performed using hapflk release 1.3 (Fariello et al., 2013). Principal component analysis (PCA) was performed in TASSELv5.2 to depict the population structure. Pairwise LD between the markers estimated in TASSEL along each chromosome and for all markers was used to visualize the LD decay according to the method described by Hill and Weir (1988), which was later modified by Remington et al. (2001). The analysis was performed in R environment (R Core Team, 2019) for each chromosome separately and heatmaps for pairwise LD per chromosome was generated using TASSEL v5.2.

### 2.3 Whole genome scan for selection sweeps

The subpopulations defined by STRUCTURE program were used for *F_ST_* analysis based on the pure drift model proposed by Nicholson et al. (2002) in R v3.4.2 as illustrated by Porto‐Neto, Lee, Lee, and Gondro (2013). Allele frequency for each variant was estimated within each subpopulation and in the whole population. The squared deviation of frequency for each subpopulation were divided by the variance of allele frequencies in the population to estimate the *F_ST_* values. Smoothing of the *F_ST_* values was performed using R‐package Lokern (Herrmann & Mächler, 2016) as suggested by Porto‐Neto et al. (2013). Variants and regions showing three standard deviations smoothed *F_ST_* values beyond average were declared as significantly under selection and therefore SS.

HapFLK statistics were estimated using hapFLK v1.3 software available at https://forge-dga.jouy.inra.fr/projects/hapflk and using a methodology described by Fariello et al. (2013). Briefly, Reynolds distances between lines were estimated using midpoint as outgroup and converted into a kinship matrix using an R script supplied with the hapFLK v1.3 package (Fariello et al., 2013). We ran hapFLK per each chromosome using the genotypes and kinship matrix assuming 15 clusters in the fastPHASE model, before the hapFLK statistic was computed as the average across 10 expectation maximization runs to fit the LD model. The P‐values for hapFLK statistics were estimated using the “scaling_chi2_hapflk.py”‐ Python script supplied at the website (https://forge-dga.jouy.inra.fr/projects/hapflk; 28 Apr. 2019). The negative log p‐value (*−logP*) for each variant was plotted using “ggplot2” package in R environment. The variants with P‐value less than 0.01 [*−logP* > 2] were declared as significant.

The coordinate of markers or selected regions were used to retrieve the genes from IWGSC v1.0 genome assembly (Appels et al., 2018). The literature was mined for evidence of known loci and genes for agronomic or quality traits. Nucleotide sequences of potential candidate genes were retrieved from NCBI (https://www.ncbi.nlm.nih.gov/), and their oligo primer sequences, retrieved from GrainGenes database (https://wheat.pw.usda.gov/cgi-bin/GG3/browse.cgi), were blasted against the wheat genome assembly to localize their position in relation to the regions we identified. This facilitated to form potential hypotheses why these regions were under selection.

### 2.4 Agronomic assessment

A total of 392 out of 436 entries were used in field testing in 2017–2018 and 2018–2019 seasons in Indiana. The experiment was planted in randomized incomplete block designs consisting of seven incomplete blocks. Each incomplete block consisted of 64 plots including 56 entries and 8 replicates of a common check ‘PU212’. Each plot was ∼3 m wide by 1 m long (12 ft by 4 ft). In the 2017–2018, one replication of a randomized incomplete block design experiment was planted at the Agronomy Center for Research and Extension (ACRE), 40.47° N, 86.99° W, elevation 185 m, West Lafayette, IN. In the 2018–2019 season, two replicates of randomized incomplete block design experiments were planted in ACRE and only one replicate was planted in Romney, IN, 40.3° N, 86.9° W, elevation 225 m.

Days to heading (DH), plant height (PH), test weight (TW) and grain yield (YLD) were recorded from all trials and plots. Yield components i.e., number of spikes per unit area (NS) and number of kernels per unit area (NK) were collected from ACRE in 2017–2018 and from Romney in 2018–2019 experiments. DH was recorded when more than 50% of the plants in each plot showed completely emerged heads from boots at Zadoks stage 59. PH in centimeters (cm) was measured from the base of the plants up to the top of the spikes (excluding awns) at physiological maturity from four random spots in each plot and were then averaged. YLD (ton ha^−1^) and TW (kg m^−3^) data were collected from each plot, while seed weight was adjusted to 13% moisture. Two adjacent rows of 50 cm each were cut and using for measuring NS and NK.

### 2.5 Statistical analysis of field data

For data analysis we first used lme4 (Bates et al., 2015) package in R v3.6.1 environment (R Core Team, 2019) as done by (He et al., 2016; Huang et al., 2016), and fitted a mixed model by taking genotypes as fixed effect and incomplete blocks as random effects per each experiment. Then, ls means per trait and sets were estimated. In follow‐up analysis, both experiments and ls means of lines were taken as random effect to extract variance components for estimation of broad sense heritability (*H^2^*) on entry mean basis. In this stage, the following model was fitted:

(1) $$Y_{i_{j}}=\mu +Line_{i}+Set_{j}+\varepsilon_{i_{j}}$$

where $Y_{i_{j}}$ is the observed phenotype, μ is the overall mean, $Line_{i}$ is the random effect of the i^th^ line, $Set_{j}$ is the random effect of j^th^ replicate, and $\varepsilon_{i_{j}}$ is the random error term. Then, using the variance components estimated from Equation 1, broad sense heritability (*H^2^*) on entry mean basis was estimated as follows:

(2) $$H^{2}=\frac{\sigma^{2}g}{\sigma^{2}g+\sigma^{2}e/r}$$

where $\sigma^{2}g$ is the genotypic variance and $\sigma^{2}e$ is the error variance, and r is the number of replicates from which the traits were evaluated. Best Linear Unbiased Estimates (BLUEs) of each line was estimated by setting genotype as fixed effects in Equation 1. Because the experimental design was incomplete, we also estimated heritability by the method described by Cullis, Smith, and Coombes (2006) and suggested by Piepho and Möhring (2007) for unbalanced data using the following equation:

(3]) $${H^{2}}_{c}=1-\frac{{\bar{v}}_{BLUP}}{2\sigma^{2}g}$$

where ${\bar{v}}_{BLUP}$ is the mean variance of all pairwise difference of BLUPs.

### 2.6 Genome‐wide association studies and genomic prediction

GWAS was performed using FarmCPU package (Liu, Huang, Fan, Buckler, & Zhang, 2016) in R v 3.6.1. The first four principal components were used to control the population structure and the markers were filtered to have minor allele frequency of more than or equal to 0.05. Student's t‐test was used to test the significance of major loci for each trait. That is the statistical comparison between the means of two groups of homozygous individuals in the population.

In GWAS procedure for any given trait, we included those major genes that showed significant effect in t‐test as fixed effect covariate. In this manuscript, we listed MTAs that were identified by the threshold of *−logP* ≥ 4. The MTAs located on the same chromosome were resolved into unique regions by estimating pairwise LD between pairs of markers and picking only one representative markers from groups that had LD ≥ 0.60. The effect of each MTA was estimated as the difference between the means of two groups of homozygous individuals for that marker in the population. The coefficient of determination (R^2^) of the markers was estimated by fitting an ordinary least square regression using phenotype as the response variable and the marker as independent variable.

For grain yield only, we also performed a five‐fold cross validation of genomic prediction using GBLUP model for 500 cycles in rrBLUP package in R to evaluate the accuracy. Accuracy of genomic prediction was evaluated as correlation between the genomic‐estimated breeding value and BLUE divided by the square root of heritability.

## 3 RESULTS AND DISCUSSION

### 3.1 Marker discovery

Next generation sequencing of reduced libraries resulted in > 696 million reads for the 436 germplasm. The reads were merged into 1,278,152 unique tags by TASSEL5 GBS v2 pipeline. Of 1,278,152 unique tags, 1,157,312 (90.55%) tags were mapped to the IWGSC Refseq v1.0 assembly (Appels et al., 2018) while the rest of them were not mapped. After variant calling, 169,486 SNPs were identified. Only 14,907 SNPs were retained after filtering for MAF and missing data.

### 3.2 Sub‐genome B revealed the greatest genetic polymorphism

The B sub‐genome revealed the greatest number of SNPs 6,546 (44%), whereas sub‐genomes A and D showed 1270 (37%) and 1,015 (17%), SNPs (Supplemental Figure FS1). Similar relative marker numbers were observed in other studies. For example, the majority of ∼47,000 SNPs identified using the 90K SNP chip array in wheat reported by Wang et al. (2014) was located on the B sub‐genome (50%), while and the D sub‐genome revealed the least number of SNPs (15%). Similarly, in a wheat re‐sequencing project conducted by Rimbert et al. (2018) using 8 wheat lines with diverse origins, 49% of the total 3.3 million SNPs were located on the B sub‐genome while only 10% located on the D sub‐genome. The occurrence of smaller number of SNPs on the D sub‐genome is most likely due to the limited D‐genome diversity contributed by few accessions of *Ae. tauschii* followed by a limited gene flow from *Ae. tauschii* to hexaploid wheat (Cox, 1997). In our study, the three chromosomes with the largest numbers of polymorphism were 2B with 1,611 (25% of the B sub‐genome), 7A with 1,270 (23% of the A sub‐genome), and 7D with 1,015 (41% of the D sub‐genome) SNPs (Supplemental Figure FS1).

### 3.3 The linkage equilibrium extent and decay varied among chromosomes

Across the entire genome, the LD decayed in half from the initial value of *r^2^* = 0.23 over a physical range of 8.79 megabase pairs (Mbp). However, chromosomes varied considerably in their LD decay behavior. For example, the fastest LD decay was observed within 642 Kbp for chromosome 4D. Substantially slower decay rates were observed within ranges 763 Mbp for chromosome 2B and 94.3 Mbp for chromosome 7D (Supplemental Table TS1). The highest number of SNPs and extended LD blocks, shown by LD heatmaps in Supplemental Figure FS2, on chromosome 2B and 7D are potentially due to alien introgression in these chromosomes from wild relatives of wheat. Chromosome 2B harbors an alien introgression from *Triticum timopheevi*, which is present in ∼40% of the individuals in this population. This region harbors the stem rust resistance gene *Sr36* (Allards & Shands, 1954; Tsilo, Jin, & Anderson, 2008). Jin and Singh (2006) evaluated the stem rust response in the US wheat cultivars and postulated that the resistance in soft winter wheat was primarily due to *Sr36* gene. The pairwise LD between all markers from chromosome 2B and KASP marker for *Sr36* (Tsilo et al., 2008) revealed that out of 1,611 SNPs on chromosome 2B, 864 located within a ∼654 Mbp (from 90.4 to 744.3 Mbp) region, showed high (*r^2^* > 0.7) LD with the KASP marker representing *Sr36*. In contrast, the average pairwise LD of the *Sr36* KASP marker with SNPs outside this region was as low as *r^2^* = 0.10, suggesting the extended LD in 2B is due to this introgression.

Chromosome 7D harbors potentially three different high‐LD islands (Supplemental Figure FS2), where one of these regions is pericentromeric and the other two are located on the opposite arms. The breeding history of this population shows alien transfer of the Barley Yellow Dwarf Virus resistance gene (known as *Bdv3*) from tall wheatgrass *Thinopyrum intermedium* (Sharma et al., 1995), possibly residing the distal end of the long arm of chromosome 7D (Crasta et al., 2000). Genotyping for *Bdv2/3* showed that 197 and 226 individuals were homozygous for positive and negative alleles, while 13 individuals were heterozygous. Out of 1,015 SNPs on chromosome 7D, 245 covering a ∼106 Mbp genomic region on the distal end of 7D showed strong LD (*r^2^ *> 0.73) with *Bdv2/3* markers (Supplemental Figure FS2), suggesting that this high‐LD island harbors the *Bdv2/3* locus. The remaining two islands could be associated with two separate alien introgressions. A region of 7E chromosome from *Thinopyrum intermedium*, a wild relative of wheat, which consists FHB resistance locus *Qfhs.pur‐7EL* (or so‐called *Fhb7*) was transferred to hexaploid wheat (Guo et al., 2015, Shen & Ohm, 2007). Similarly, another alien introgression in chromosome 7D is from wheat relative *Thinopyrum ponticum* (Bariana et al., 2007) and present in ‘Wheatear’, an Australian spring wheat variety, that consists of stem rust and leaf rust resistance gene block *Sr25/Lr19*.

### 3.4 Population structure and pedigree analysis

**FIGURE 1:**
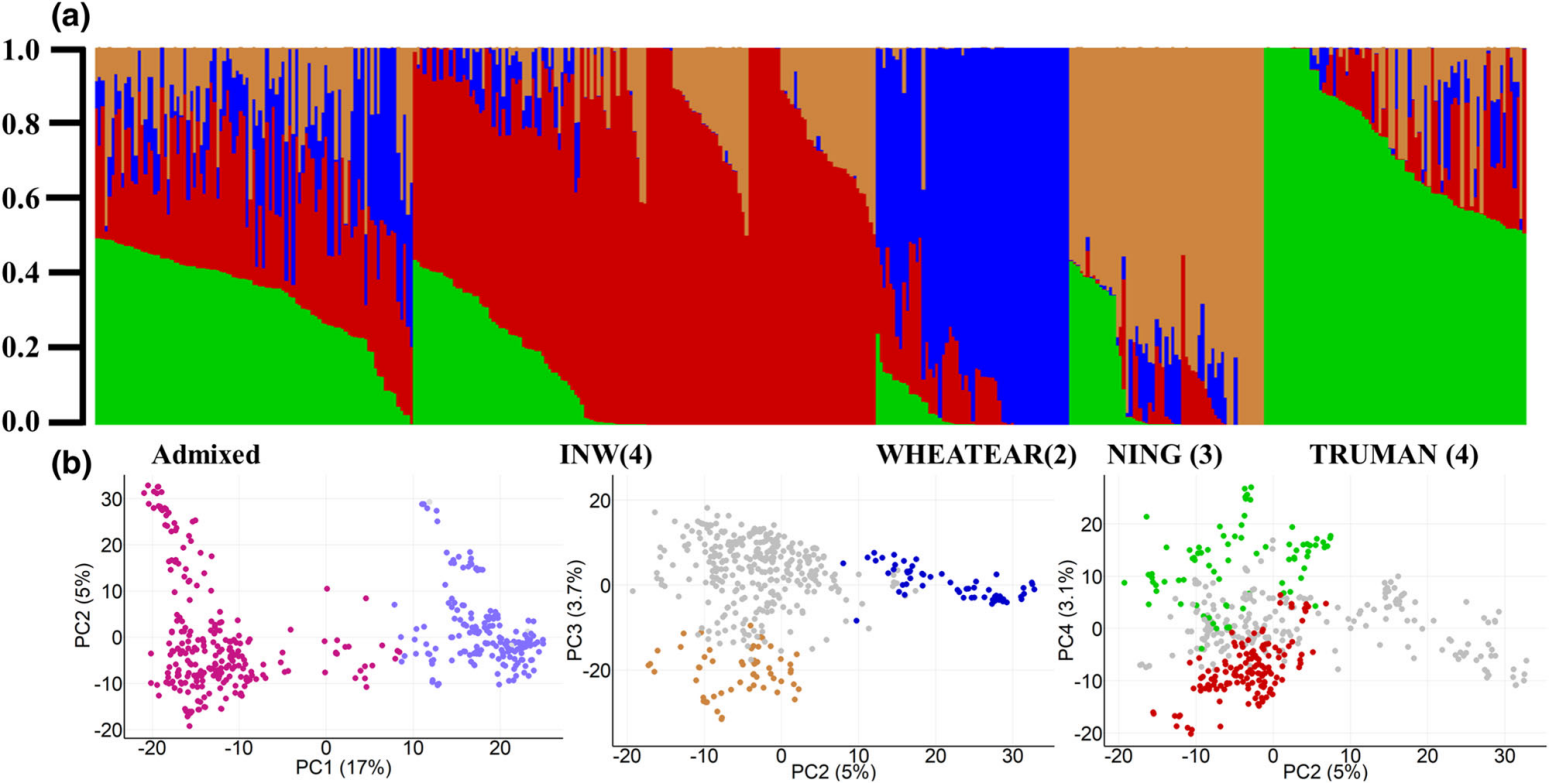
Population structure inferred by STRUCTURE software for k = 4 and principal component method. The stacked bar plot (a) shows ancestry estimates for each accession in each of the four subpopulations and in the admixture. (b) Scatterplot of principal components (PC). Scatterplot on the right shows the separation of germplasm based on 2B:2G introgression. The pink color represents individuals without the 2B:2G translocation and the blue color shows individuals with the 2B:2G translocation. The two scatterplots on middle and right show the relationship between four principal components and sub‐populations inferred from STRUCTURE. The color of these scatterplot was matched the color of sub‐populations derived from the STRUCTURE analysis for ease of comparison

For STRUCTURE analysis, the maximum delta K value reached at K  =  4, indicating that a four‐subpopulation model fits best to the genotypic data. A graphical display of the ancestry estimates for each accession in each subpopulation is shown in Figure 1A. A membership coefficient > 0.5 was used to assign lines to a specific subpopulation. Individuals that did not have membership coefficient > 0.5 for any of the subpopulations were labeled as ‘admixed’. The pedigree of lines with 99.9% ancestry coefficient in each subpopulation was retrieved to assign the subpopulation to the most widely known or used genetic background. The four subpopulations identified were named after the widely known germplasm in the group.

In subpopulation 1 (80 entries), eight entries showed 99.9% ancestry coefficient. Three of these eight lines were derived from a cross designated as ‘04606RA1’. Another three lines were derived from a cross designated as ‘0527A1’. The lines from ‘04606RA1’ are derivatives of ‘TRUMAN’ when it was crossed with ‘961341A3‐1‐4‐6’. The lines with ‘0527A1’ pedigree are progeny of cross ‘04617A1’ × ‘04688A1’ lines. Similarly, the remaining two lines are ‘07469A1‐6‐1‐1’ and ‘TRUMAN’. This subpopulation was named TRUMAN. Truman is a soft red winter wheat variety developed at Missouri Agricultural Experiment Station and has been used as a native source for FHB resistance for several years (McKendry, Tague, Wright, Tremain, & Conley, 2005; Petersen et al., 2017). In subpopulation 2 (59 entries), 16 entries are from the cross ‘10644C1’ (‘10143’ × ‘P25R62’) that showed 99.9% ancestry coefficient. The parent line ‘10143’ itself, is a progeny of ‘07469A1‐10‐1’ × ‘Wheatear’. This subpopulation was named WHEATEAR. Wheatear is an Australian wheat variety harboring the *Sr25*/*Lr19* resistance gene block from *Thinopyrum ponticum* (Bariana et al., 2007; Zhang et al., 2005). KASP assay for *vrn1* gene for all 5 homoeologous group chromosomes showed that 44 individuals (∼76%) from this group possessed at least one spring type allele suggesting that this group is segregating for spring growth habit alleles possibly inherited from Wheatear. Ten out of 59 entries in subpopulation 3 showed 99.9% ancestry coefficient. Of the 10 lines, four lines are progeny of a cross designated as ‘0527A1’. Two lines are progeny of a cross designated as ‘0570A1’, while other lines are progeny of four other crosses designated as ‘04704A1’, ‘0537A1’, ‘0858A1’, ‘0925A1’. According to their pedigrees, all these lines have two common ancestors ‘Ning‐7840’ and ‘Clark’ in their recent breeding history. This subpopulation was named NING. Ning‐7840 is a Chinese hard red cultivar that has been used as an exotic source of FHB resistance in breeding programs (Drake, 2004). Nine lines out of 141 entries in subpopulation 4 showed 99.9% ancestry coefficient. All the lines are from a cross designated as ‘HORM45’. They are double haploid progeny produced from a cross between ‘INW0411’ and ‘INW0412’. This subpopulation was named INW. ‘INW0411’ and ‘INW0412’ are soft red winter wheat cultivars developed by Purdue University. The cultivar ‘INW0411’ has excellent milling properties and medium gluten strength. ‘INW0411’ is the progeny of ‘96204A1‐12’ × ‘9723RE’ cross. The parents of ‘96204A1‐12’ are ‘FREEDOM’ and ‘92829A1‐1‐5‐1‐4‐6’. The parents of ‘9723RE’ are ‘Goldfield’ and ‘INW9824’. The cultivar ‘INW0412’, is known for its high normalized test weight and medium strong gluten and a progeny of ‘ACC3130_HYAPEI57‐2’ × ‘PATTERSON’ cross.

In the genomic population tree, (Supplemental Figure FS3), TRUMAN, INW, and admixtures clustered together in a monophyletic group, indicating these population are relatively closer to each other, probably due to the adaptation of germplasm to the U.S. soft red winter wheat growing regions. STRUCTURE revealed that TRUMAN and INW sources dominated the ancestry coefficient in the ADMIXTURE population, and that is why the admixed subpopulation is immediately grouped to the TRUMAN and INW subpopulations (Figure 1A). WHEATEAR and NING subpopulations are distant from each other and distant from the other subpopulations, possibly because of their country of origins.

The majority of genotypic variations was explained by only the first four principal components, with only minimal marginal increase after that. The first four PCs together explained 28.8% (PC1 = 17%, PC2 = 5%, PC3 = 3.7%, and PC4 = 3.1%) of the total genotypic variation. The first principal component (PC1), explaining 17% of the total variation, distinguishes germplasm are contrasting for presence or absence of the 2B:2G translocation. Based on presence/absence of *Sr36/Pm6* regions, the individuals that carry 2B:2G translocation were almost perfectly separated by PC1 from the individuals that did not (Figure 1B). Similar results were reported in soft red winter wheat grown in Virginia (Ward et al., 2019). We did not see such separation in the population using STRUCTURE. This is partly due to running of PCA on all SNPs while STRUCTURE on only reduced set of SNPs. PC2 and PC3 separated WHEATEAR and NING subpopulations from rest of the germplasm (Figure 1B). Similarly, PC4 separated TRUMAN and INW subpopulations from the rest of the germplasm (Figure 1B).

### 3.5 Whole genome scan for selection sweeps

**FIGURE 2:**
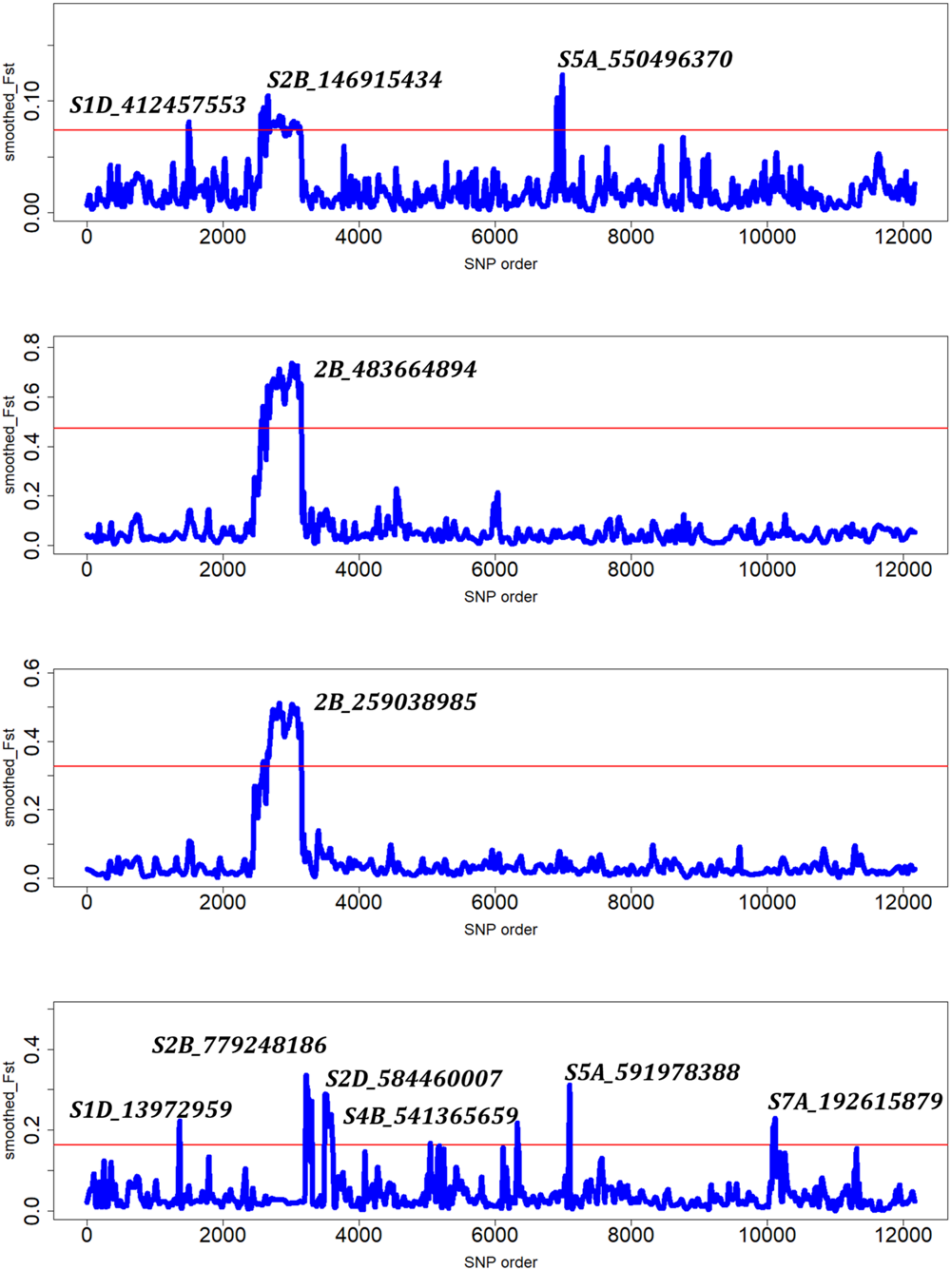
Whole genome scans for positive selection by using FST method. The x‐axis shows the physical position of the variants. The horizontal red lines represent the threshold (average plus three orders of the standard deviation) for declaring significance

Using *F_ST_* approach and comparing the subpopulations identified via STRUCTURE analysis, 12 regions on eight chromosomes showed SS (Table 1; Figure 2). Similarly, using hapFLK statistics, four genomic regions including two on chromosome 2B, one on 3B and one on 5B were identified to be significantly selected (Supplemental Figure FS4). Between the smoothed *F_ST_* and the hapFLK approach, only one significant genomic region on chromosome 2B was overlapping.

**TABLE 1:** Genomic regions detected to be under selection by smoothed *F_ST_* and hapFLK statistics. The region start and end were determined by the position of flanking markers that were above the significant threshold

Chrom.	Region Start^a^	Region End^b^	Peak location^c^	Subpopulation	Number of genes	Associated region	References
1D	411731821	414719517	412457553^d^	TRUMAN	36	*Glu‐D1*	Shewry et al., 2003
	12823191	15336900	13972959^d^	INW	23	*LT*	Båga et al., 2006; Fowler et al., 2016
2B	319090	46602138	24851652^e^	Whole Panel	828	Unknown	
	101072502	570883707	146915434^d^	TRUMAN	2673	*2B:2G*	Tsilo et al., 2008
	53577523	723331990	483664894^d^	NING	3688	*2B:2G*	Tsilo et al., 2008
	53986622	655597674	259038985^d^	WHEATEAR	3684	*2B:2G*	Tsilo et al., 2008
	775333503	800920106	779248186^d^	INW	413	Unknown	
	763445978	800920106	776406624^e^	Whole Panel	563	Unknown	
2D	580117352	617097449	584460007^d^	INW	520	Grain protein content	Mahjourimajd et al. (2016); Prasad et al., 2003
3B	6181603	14025949	10170821^e^	Whole Panel	162	*FHB1*	Su et al. (2019)
	711349262	753951947	711642065^d^	INW	421	Grain Number	Sukumaran, Lopes, Dreisigacker, & Reynolds, 2018
4B	535092535	553506651	541365659^d^	INW	107	TKW	Lozada, Mason, Sukumaran, & Dreisigacker, 2018
5A	503873008	552747815	550496370^d^	TRUMAN	546	*FR‐A2*	Miller et al., 2006; Vágújfalvi et al., 2005
	583031341	595158840	591978388^d^	INW	171	*VRN‐A1*	Yan et al., 2003
5B	550910513	585008944	556185926^e^	Whole Panel	368	*VRN‐B1*	Yan et al., 2003
7A	149426415	206581766	192615879^d^	INW	425	*VRN3*	Yan et al., 2006

a The position of left flanking identified SNP based on *F_ST_* or p‐value of hapFLK test above the significant criteria.

b The position of right flanking identified SNP based on *F_ST_* or p‐value above the significant criteria.

c The position of the SNP with the maximum *F_ST_* in each region or −log(p) value of hapFLK test.

d The region was identified as significantly selected using smooth *F_ST_* method.

e The region was identified as significantly selected using haFLK method.

The regions on chromosome 2B from TRUMAN, NING, and WHEATEAR subpopulations were overlapping (Table 1; Figure 2). Based on smoothed *F_ST_*, the NING group seems to have the largest region on chromosome 2B, spanning roughly over 669 Mbp, harboring the 2B:2G translocation. TRUMAN and WHEATEAR groups showed subsets of the 2B region identified in the NING group (Table 1). In INW group, a second region of 2B was identified that falls ∼52 Mbp downstream of the 2B:2B translocation. This second region is between 775.3 and 800.9 Mbp, which was also supported by the hapFLK method (Table 1).

Regions on chromosome 1D and 5A were identified in TRUMAN and INW groups (Table 1). In TRUMAN group, the region on chromosome 1D spans from 411.7 to 414.3 Mbp, while the region in INW group covers from 12.8 to 15.3 Mbp (Table 1), indicating that these are two distinct regions physically located ∼398 Mbp apart. The significantly selected regions on chromosome 5A in TRUMAN and INW groups are 30 Mbp apart (Table 1; Figure 2). In TRUMAN group, the 5A selected region spans from 503.9 to 552.7 Mbp whereas in INW group, it ranges from 583 to 595.2 Mbp. Other genomic regions on chromosomes 2D (580.1 to 617.1 Mbp), 3B (711.3 to 754 Mbp), and 4B (535.1 to 553.5 Mbp) were also found only in INW group (Table 1; Figure 2).

The availability of wheat reference genome IWGSC RefSeq V1.0 (Appels et al., 2018) enabled us to retrieve the candidate genes within each region. Most of the regions identified in this study ranged from 2.5 Mb (on 1D) to 669 Mbp (on 2B), including from 23 genes to 3688 genes. Therefore, pinpointing the genes under selection is speculative. We searched the literatures to find QTLs with meaningful agronomic implications around those regions as existing support; however, further evidences are required to validate these results.

Two evidence suggest that the 1D region (12.8 to 15.3 Mbp) identified in INW group could be involved in low‐temperature tolerance (LT). Båga et al. (2007) identified two QTL on chromosomes 5A and 1D for LT using ‘Norstar’ (high LT) × ‘Manitou’ (low LT) population. In the region identified in this study on 1D between microsatellite markers BARC159 and BARC169, covered from 12,203,610 bp to 328,441,452 bp is located within the region we identified. Moreover, the wheat gene Transducin/WD40 repeat protein (TraesCS1D01G034200) located in the region shows 79.6% identity to Arabidopsis HOS15 (NCBI refseq: NP_178182.1), which is also a WD40 repeat protein and has been shown to be important for cold tolerance where *hos15* mutant *Arabidopsis* plants were hypersensitive to freezing (Zhu et al., 2008). The 1D region (411.7 to 414.3 Mbp) of TRUMAN group consists of 36 genes, which includes a high molecular weight glutenin subunit *Glu‐D1* gene controlling gluten quality of the wheat (Anjum et al., 2007). The 2D region (580.1 to 617.1 Mbp) of INW group consists of 520 genes. Near this region, two grain protein content QTL were reported. Prasad et al. (2003) mapped QTL for grain protein content linked to wms1264 located from 577,440,661 to 577,440,823 bp on chromosome 2D. Another grain protein QTL was found between markers located from Ex_c2115_3369 (435,045,172 bp) and Kukri_c9145_1322 (571,058,395 bp) on chromosome 2D (Mahjourimajd et al., 2016).

The region identified by hapFLK on chromosome 3B is most likely selected for *Fusarium* head blight resistance because it includes *Fusarium* head blight resistance locus *Fhb1*. The *Fhb1* locus from Sumai 3 cultivar is the most widely used source of FHB resistance (Bai, Kolb, Shaner, & Domier, 1999; Waldron, Moreno‐Sevilla, Anderson, Stack, & Frohberg, 1999). Su et al. (2019) performed fine mapping of *Fhb1* locus and showed that *TaHRC*, a gene that encodes a putative histidine‐rich calcium‐binding protein is responsible for FHB resistance. The *TaHRC* gene (GenBan ID: CBH32655.1) is located from 8,526,628 to 8,529,572 bp, which resides inside 6.2–14 Mbp region identified in this study.

Four regions were identified on chromosomes 5A, 5B, and 7A with implication in vernalization requirements and freezing tolerance. Vernalization and freezing tolerance are controlled by several loci i.e., *Vrn1* (Yan et al., 2003), *Vrn2* (Yan et al., 2004), and *Vrn3* (Yan et al., 2006) and *Fr‐A2* (Vágújfalvi et al., 2005). The *Fr‐A2* locus is located 30 cM proximal to *Vrn1* and reported to be consisting of a cluster of eleven or more C‐repeat Binding Factors (CBF) genes (Miller, Galiba, & Dubcovsky, 2006; Vágújfalvi et al., 2005). The CBF genes are member of AP2/EREBP transcription factor family that are essential for freezing tolerance (Thomashow, Gilmour, Stockinger, Jaglo‐Ottosen, & Zarka, 2001). We retrieved gene sequences of *Vrn‐A1* (GenBank ID: GQ451810.1), *Vrn‐B1* (GenBank ID: HQ130483.2) and *Vrn‐2* (GenBank ID: AAS60251.1) from NCBI and BLAST them against IWGSC Refseq v1.0 genome. The *Vrn‐A1* and *Vrn‐B1* genes were located 587,423,705 to 587,423,039 bp and 698,178,816 to 698,179,052 bp on chromosomes 5A and 5B respectively. In addition, we located 17 copies of CBF genes in the IWGSC V1.0 reference genome from 522,066,602 to 523,616,451 bp on chromosome 5A that is known to be associated with *Fr‐A2* locus.

The 5A region (503.9–552.7 Mbp) of TRUMAN group includes the 17 copies of CBF genes. Similarly, the 5A region of INW group spans from 583 to 595.2 Mbp that includes *Vrn‐A1* gene. The 7A region (149.4 to 206.6 Mbp) consists of *FLOWERING LOCUS T/TERMINAL FLOWER 1*‐like protein (TraesCS7A01G229400, 199,671,796‐199,673,399 bp), a gene that has homology to the *Vrn‐B3* candidate gene (Yan et al., 2006). This finding suggests that the individuals in TRUMAN and INW group were potentially selected for freezing tolerance.

This study showed that of the 13 significantly selected regions, 5 loci (*LT*, *Fr‐A2*, *Vrn‐A1*, *Vrn‐B1*, and *Vrn3*) are involved in environmental adaptation such as flowering time and winter hardiness. Other loci were associated with disease resistance such as *Sr36* and *Fhb1* and end‐use quality traits such as *Glu‐D1* and *GPC*. Regions associated with vernalization also showed SS in previous studies. Cavanagh et al. (2013) used genome wide markers to identify genomic regions selected during domestication and post‐domestication. They observed genetic differentiation for *Vrn‐A1*, *Vrn‐B1* loci but not for photoperiod regulation genes. The evidence of selection on the region containing *Vrn‐A1* gene was also reported by Gao et al. (2017) in comparison of wild relatives, landraces and modern cultivars of wheat.

### 3.6 Trait correlations and heritability

The trait distributions, mean, and range are provided in Supplemental Table TS2 and Supplemental Figure FS5. The germplasm headed as early as 131 days to 147 days (after January 1). The average number of spikes was 711 spikes m^−2^, with a maximum of 1081 spikes m^−2^. Grain yield ranged from 1 to 9, and averaged ∼4.4 t ha^−1^. There was moderate negative correlation between DH and yield‐related traits: TW, NS, and NK as well as the YLD (r = −0.44, −0.23, −0.14, and −0.38 respectively). This could be due to recurrent selection for early flowering and high yielding cultivars in the past. PH showed no or very low correlation with DH, NS, and NK, suggesting that these can improved in an independent manner. There was meager positive correlation between YLD and PH (r = 0.27). All the four yield related traits were positively correlated with each other as expected. TW was highly correlated with NS and YLD (0.63 and 0.64 respectively). For the rest of the trait‐combinations, the coefficient of correlation ranged from 0.19 between YLD and NS to 0.35 between YLD and NK.

Broad‐sense heritability based on entry mean (*H^2^*) was moderate for NK (0.33), NS (0.42), and YLD (0.53) but relatively higher for TW (0.64), PH (0.83) and DH (0.72) (Supplemental Table TS6). Heritability based on Cullis et al. (2006) method (*H^2^_c_*) was consistently lower than *H^2^* estimates although trend was similar (Table 3). Schmidt et al. (2019) showed that heritability based on entry mean method overestimates in comparison to heritability estimates using method described by Cullis et al. (2006) especially in unbalanced agriculture trails similar to this study.

### 3.7 Diversity and effects of major genes on agronomic traits

*Rht‐B1b* allele that reduces height showed the highest frequency of 0.89. The *Rht‐B1* gene was highly significant for PH and the Rht‐B1b allele reduced the plant height by ∼10 cm (Table 2). In addition, it decreased grain yield by −0.34 t at p‐value 0.048. Conversely, Hayat et al. (2019) reported a 5% yield‐increasing effect for the *Rht‐B1b* allele in an SRW wheat population. Only four individuals were homozygous for *Rht‐D1b* allele, and only two lines were double mutants at *Rht1* loci. We did not evaluate allelic effects for these due to low frequency.

**TABLE 2:** T‐test significance level and marker effects for major loci on agronomic traits. Allelic effects are estimated as the difference between mean of homozygous individuals with mutant and wild allele for each major locus. Numbers below the major loci names represent the number of homozygous lines with mutant and wild allele respectively

		Rht‐B1	Ppd‐A1	Ppd‐B1	Ppd‐D1	vrn‐A1	1RS.1BL	Sr36/Pm6
Traits	Units	(341:35)	(171:188)	(94:298)	(241:133)	(113:249)	(133:232)	(151:238)
Days to heading	Julian days	−0.1	−0.33	−0.32	−0.83^***^	0.27	0.8^***^	−0.27
Plant height	cm	−9.69^***^	−2.65^**^	−3.44^***^	−2.03^*^	−0.21	−1.28	−1.7^*^
Test weight	kg m^−3^	−12.84	4.04	22.25	−29.03^*^	−28.76^*^	−42.4^***^	21.25
Grain yield	t ha^−1^	−0.34^*^	−0.1	0.07	−0.30^*^	−0.16	−0.19	0.16
Spikes per unit area	spikes m^−2^	−31.71	13.93	13.67	3.43	−34.17^*^	−38.44^**^	32.67^**^
Kernels per unit area	kernels m^−2^	−658.72	−1268.64^*^	795.53	−48.35	749.48	−317.62	1.95

^*^Allelic effect is significant at the 0.05 level;

^**^Allelic effect is significant at the 0.01 level;

^***^Allelic effect is significant at the 0.001 level.

The photoperiod insensitive allele *Ppd‐D1a* was present in 64% of the population and had an accelerating effect on days to heading (by ∼20 hours) and other traits (Table 2). The *Ppd‐B1a* and *Ppd‐A1a* photoperiod insensitive alleles, present in 46% and 24% of the population, respectively, did not show any significant effect of days to flowering.

The frequency of the rye translocation 1RS.1BL was ∼39%. We observed a significant negative effect from 1RS.1BL translocation on DH, TW, and NS, but not YLD (Table 2). McKendry, Tague, and Miskin (1996) did not observe any significant yield advantage while heading was delayed in lines with the translocation. Similarly, Zhao et al. (2012) reported significant reduction of TW and kernel weight in lines possessing the translocation.

The frequency of the stem rust resistance gene *Sr36* was ∼37%. The presence of *Sr36* gene significantly reduced plant height and increased NS (Table 2). Ward et al. (2019) reported significant reduction in plant height in lines with *Sr36* genes in an SRW wheat population grown in Virginia. However, no significant effect was observed for NS by Ward et al. (2019).

### 3.8 GWAS for major agronomic traits

Association of genome‐wide markers with six agronomic traits was analyzed by using FarmCPU model. In earlier GWAS, false positives were controlled by accounting for population structure and kinship i.e., Q + K. While successful for controlling false associations,, true association could not be detected due to the confounding nature of the SNP being tested because of the way the kinship matrix accounted for relatedness. When estimating kinship, FarmCPU removes the confounding effects of kinship matrix and the SNP that is being tested and thus, provides greater power to identify true associations. Several studies have validated such increase in power to detect true associations.

Liu et al. (2016) compared the performance of seven different models including mixed linear model (MLM), compressed MLM (CMLM), and FarmCPU. In their study, they evaluated flowering time of 199 *Arabidopsis thaliana* accessions by using 250,000 SNPs. While MLM and CMLM controlled the false positives well, they failed to identify any significant SNPs at threshold of 1% Bonferroni correction. Meanwhile, FarmCPU controlled false positives while successfully identifying true associations even at 1% Bonferroni correction. Similarly, Kaler, Gillman, Beissinger, and Purcell (2020) compared eight different models in Soybean and Maize using simulations and empirical data. FarmCPU correctly identified most of the simulated or known QTL while other models either showed false positives or could not detect the significant regions.

For any GWAS analysis, we included major genes as fixed effect covariates to account for their effects and to avoid the association of the SNP markers that are correlated with the major genes. *Ppd‐D1* was significant for most of the traits: DH, PH, TW, and TKW whereas *Ppd‐B1* was only significant for PH (Table 2). Thirty‐five marker‐trait associations (MTAs) were identified at *−logP* > 4 across 13 chromosomes of wheat. After pairwise LD analysis, 35 MTAs resolved into 27 unique genomic regions. Seven of the 27 regions passed the 5% FDR threshold (Table 3). In addition, any MTA with R^2^ ≥ 5% was considered as major effect MTA.

**TABLE 3:** Summary of significant marker‐trait associations identified by FarmCPU algorithm

Traits	Locus^*^	Variants^a^	−log(p)	MAF	R^2^ (%)	Effect
DH (Julian days)	4A_495327498	T/A (276:101)	4.5	0.28	2.3	0.64
	5A_5678539	G/A (307:80)	5.3	0.21	0.3	0.28
	6A_20163710	A/C (214:161)	4.3	0.43	0.2	0.14
	6D_1572162	G/A (351:39)	4.1	0.1	0.6	0.47
	7B_8885441^*^	C/T (356:34)	11.4	0.09	11.5	2.18
	7D_71569531	G/A (246:128)	4.6	0.35	0.4	0.25
PH (cm)	1A_494799791^*^	A/C (364:23)	5.4	0.07	3.3	5.68
	3B_6981619	C/T (245:139)	5.1	0.36	0.5	1.23
	4A_41973900^*^	C/G (331:48)	7.3	0.14	3.8	3.78
	5B_455980623^*^	A/G (299:81)	5.5	0.22	2.3	2.94
	6A_27020312	G/T (358:7)	5.1	0.05	3.8	7.55
	6A_420391165^*^	C/G (270:111)	7.9	0.3	8.1	4.96
	6B_155468149	G/A (360:32)	5.3	0.08	2.6	4.55
	6B_603911083	C/T (197:175)	4.4	0.47	1.8	2.11
	7B_714780111	G/A (331:57)	5.1	0.15	3.4	4.0
	7D_4501942	C/T (353:18)	5.3	0.07	9.2	10.34
TW (kg m^−3^)	2A_719404657	C/T (329:55)	4.9	0.15	0.6	26.48
	3B_487539976	A/C (189:183)	4.2	0.49	1.7	29.57
	4B_3295544^*^	C/T (336:20)	5.1	0.09	0.005	6.23
	4B_573294337	C/T(342:39)	6.2	0.11	4.4	79.11
	4B_612333025	T/A (307:68)	5.4	0.2	0.5	16.59
	5A_47458032	C/T (211:161)	4.7	0.44	0.2	10.46
NS (spikes m^−2^)	1A_549422405^*^	A/G (368:22)	5.8	0.06	6.2	130.32
	1D_443717338	A/G (344:6)	4.9	0.07	6.9	93.81
	3B_419241935	G/A (198:187)	4.3	0.49	3.9	48.34
	5B_679675578	T/C(309:71)	5	0.2	2.5	48.26
NK (kernels m^−2^)	5A_456261322	C/T (348:39)	4.1	0.11	12.4	5180.78

DH: days to heading, PH: plant height, TW: test weight, YLD: yield, NS: number of spikes per square meter, NK: number of kernels per square meter.

^*^The markers are significant at 5% False discovery rate (FDR) adjusted p‐values.

a The nucleotides underlined are considered the favorable alleles. For PH and DH, allele reducing phenotypes were considered favorable whereas for TW, YLD, NS, and NK, allele increasing the phenotypes were considered favorable.

**FIGURE 3:**
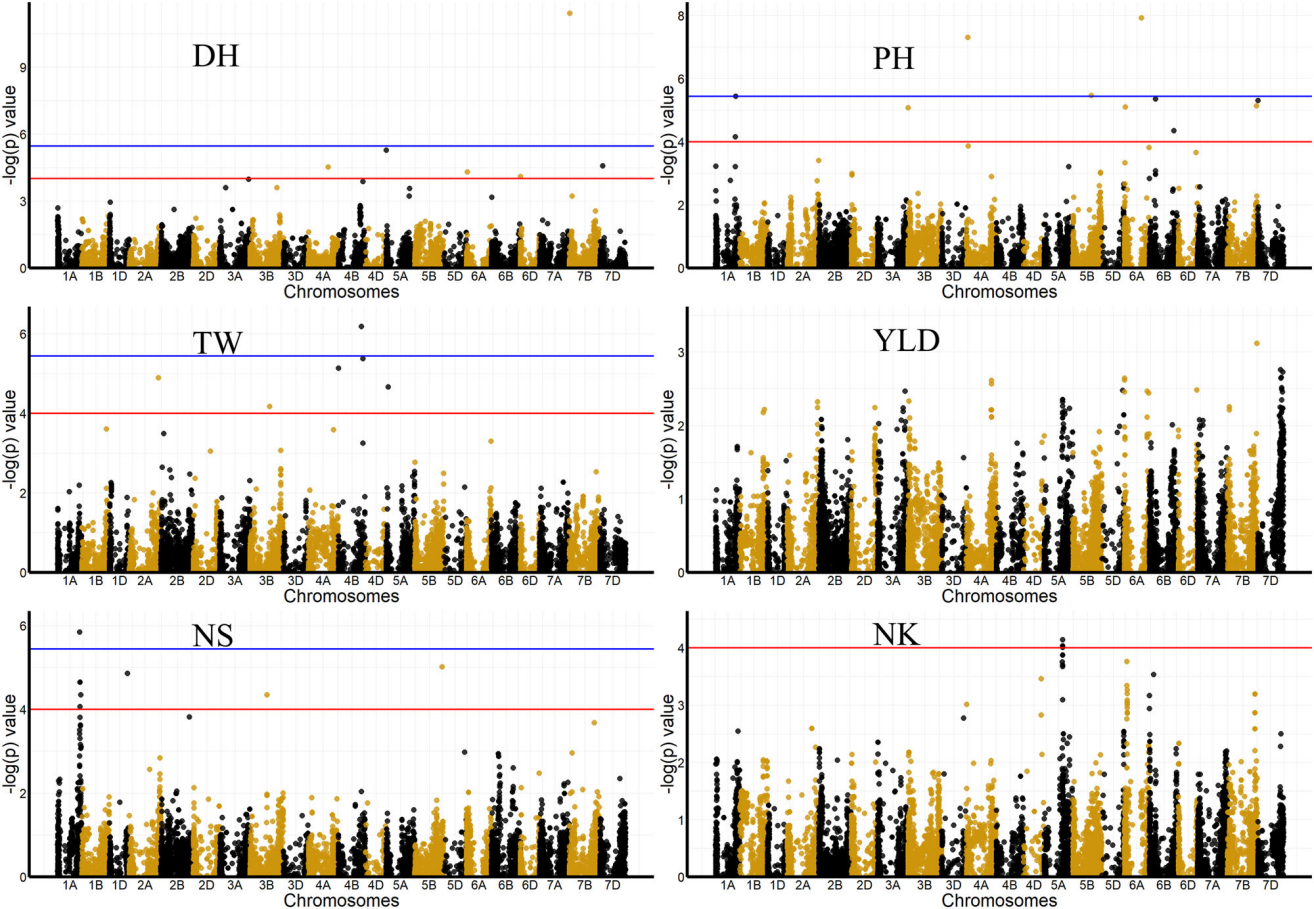
Manhattan plots of GWAS for days to heading (DH), plant height (PH), number of spikes per square meter (NS), and number of kernels per square meter (NK). The red and blue horizontal lines correspond to the −*logP* value of 4.0 and 5% FDR threshold, respectively

*Ppd‐B1* and 1RS.1BL translocation were used as fixed effect covariate for GWAS analysis of DH (Table 2). Six independent regions were identified on 4A, 5A, 6A, 6D, 7B, and 7D for DH, of which only the variant on chromosome 7B (7B_8885441) was identified at 5% FDR threshold (Table 3; Figure 3). This variant explained the highest amount of variation (11.5%) for DH, where the early heading allele (T) reduced DH by more than two days (Table 3). Chromosome 7B harbors *Vrn‐B3* locus that plays an important role in vernalization requirement and controlling flowering (Yan et al., 2006). The gene resides within 9702541 to 9703732 bp and is approximately 800 kb away from the MTA and well within the average LD decay rate (dropping to half‐LD within ∼3 Mbp) observed in chromosome 7B suggesting the region is associated with *Vrn‐3* gene.

The *Ppd‐A1*, *Ppd‐B1*, *Ppd‐D1*, *Rht‐B1*, and *Sr36/Pm6* loci were significant for PH and were used as fixed effect covariates in the GWAS model for PH (Table 2). Ten genomic regions were identified at *logP* > 4 on chromosomes 1A, 3B, 4A, 5B, 7B, 7D, 6A, and 6B of which four MTAs i.e., 1A_494799791, 4A_41973900, 5B_455980623, and 6A_420391165 were identified at 5% FDR threshold (Table 3; Figure 3). The variants 7D_4501942 and 6A_420391165 explained 9.2% and 8.1% of variation in PH, with height reducing effects of 10 and 5 cm, respectively (Table 3). The variant 6A_420391165 resides in the genomic region of *Rht24* that was previously identified by Spielmeyer et al., (2007) and further fine mapped by Li et al. (2015) between Xwmc256 and Xbarc103 markers (176,504,290 and 549,523,882 bp). In addition to 7D_4501942 and 6A_420391165, four other variants 1A_494799791, 4A_41973900, 6A_27020312, and 7B_714780111 explained ∼3% variation in PH variation and marker effects were 3.8 cm for 4A_41973900 and 7.6 cm for 6A_27020312 (Table 3). For TW, *Ppd‐D1*, *Vrn‐A1*, and 1RS.1BL were significant (Table 2). Using them as fixed effect covariate, six genomic regions were identified at −*logP* > 4 for TW. There were three independent regions on chromosome 4B and one each of chromosomes 2A, 3B, and 5A (Table 3; Figure 3). The variant 4B_573294337 that explained 4.4% variation of TW, with effect size of 79 kg m^−3^, was identified at 5% FDR threshold. Other studies have found QTL controlling TW (*QTW.ndsu.4B.2*) using a bi‐parental population (Kumar et al., 2016) which is only 3 Mbp away from the MTA identified in this study. The other MTA explained a very small percentage of variation in TW.

For YLD no marker trait association was identified, highlighting that larger effect QTL are not segregating within the population and in addition, the study is underpowered to detect minor effect QTL. In this situation, genomic selection can be effective to achieve higher genetic gains. In this study, when did a five‐fold cross validation of YLD over 500 repetitions, on average, prediction accuracy of 0.63 was achieved suggesting genomic selection could be an alternative strategy to MAS to improve yield.

For NS, major genes *Vrn‐A1*, 1RS.1BL, and *Sr36/Pm6* were used as fixed effect covariates. Four regions on chromosomes 1A, 1D, 3B, and 4B were identified for NS (Table 3; Figure 3). The favorable allele for variant 1A_549422405, identified at 5% FDR threshold, showed highest effect, increasing NS by 130 spikes m^−2^ and explaining 6.2% variation of NS (Table 3). A QTL responsible for number of kernels and spikes per square meter was recently identified 14 Mbp away from the region we identified. This QTL was identified in Weebill × Bacanora DH recombinant population and linked to the marker BS00009700 at physical position of 535062221 to 535062316 bp (Griffiths et al., 2015). Similarly, the variant marker 1D_443717338 explained ∼7% variation in NS with the favorable allele increasing NS by 94 spikes m^−2^. The variants 3B_419241935 and 5B_679675578 explained 2.5% and 3.9% of variation in NS, respectively. In both cases, the marker effect was ∼48 spikes m^−2^.

After accounting for large effect of *Ppd‐A1* in GWAS for NK, only one region on chromosome 5A was identified for NK at *−logP* > 4 (Table 3; Figure 3). The variant 5A_456261322 explained 12.4% variation in NK and the favorable allele is estimated to increase NK by 5,180 kernels m^−2^ (Table 3). Zhang et al. (2018) identified a significant MTA IWB1040 (437208847 bp on 5A) associated with grain weight in a population of spring wheats. Similarly, in a Pacific Northwest Winter Wheat population, Gizaw et al. (2018) identified marker IWA 3099 (48171543 bp) associated with thousand kernel weight (TKW).

With a focus on genotypic and phenotypic data, we characterized 436 germplasm for population structure and the extent of LD decay. Two chromosomes 2B and 7D showed extended low recombination regions possibly due to alien chromatins. STRUCTURE and PCA analyses revealed there are four sub‐populations in the populations. Pedigree analysis of these sub‐population suggested that two subpopulations, WHEATEAR and NING, were most likely result of introduction of exotic lines NING and WHEATEAR into the breeding program for disease resistance. The whole genome was scanned by using smoothed *F_ST_* and hapFLK methods to detect SS, where 13 regions with implications in environmental adaptation, disease resistance, and end use quality were identified. Field‐based phenotyping allowed for trait evaluation and GWAS analysis. We identified six major QTLs associated with agronomic (PH and DH) and yield‐related traits (TW, NK, and NS). We did not find any significant regions for YLD using GWAS, however, prediction of YLD showed that reliable accuracies can be achieved for yield improvement in this population.

This study helps to use large effect MTA and associated germplasm as parents in crossing nurseries with the aim to effectively pyramid the beneficial alleles. In addition, detailed characterization of population structure will also guide to optimize breeding populations for maximized diversity and heterotic patterns. Higher genetic variances can be achieved by crossing individuals from separate sub‐populations. For example, line ‘PU390’ from INW sub‐population with good YLD (7.6 t ha^−1^) and early flowering (135 days) and line ‘PU378’ from TRUMAN sub‐population with yield 6.4 t ha^−1^ and flowering ∼137 days seems to be ideal candidates as parents for making crosses as they come from different sub‐populations and show good YLD and early flowering.

## CONFLICT OF INTEREST STATEMENT

The authors declare that they have no conflict of interest.

## Supporting information


**Supplemental Figure FS1**. Distribution of SNPs across the sub‐genomes and the 21 chromosomes of hexaploid wheat.

**Supplemental Figure FS2**. The heatmap of linkage disequilibrium for chromosomes 2B (left) and 7D (right), showing extended islands of high‐LD.

**Supplemental Figure FS3**. Genomic population tree based on Reynold's distance matrix of lines, where all markers were used to derive distances.

**Supplemental Figure FS4**. Manhattan plots showing the *−logP* values based on the hapFLK method.

**Supplemental Figure FS5**. Histograms (diagonals), pairwise scatterplots (lower triangle from the diagonal of histograms), and the pairwise Pearson's correlations (upper triangle from the diagonal of histograms) are presented.

**Supplemental Table TS1**. The table shows the LD decay distances for individual chromosomes.
**Supplemental Table TS2**. Summary of descriptive statistics and heritability of the six agronomic traits.

**Supplemental Information SI2**. The phenotypic, genotypic, and the major gene covariates used for GWAS analysis are provided.

## Data Availability

The genotype and phenotype data are available in the Supplemental information.
