# Supplementary material for: Identification of regions under selection and loci controlling agronomic traits in a soft red winter wheat population
Source: Plant Genome. 2020 Jun 29;13(2):e20031. doi: 10.1002/tpg2.20031 (PMC12807307; doi:10.1002/tpg2.20031)
Supplement: Supplementary file 1 [file TPG2-13-e20031-s004.pdf]

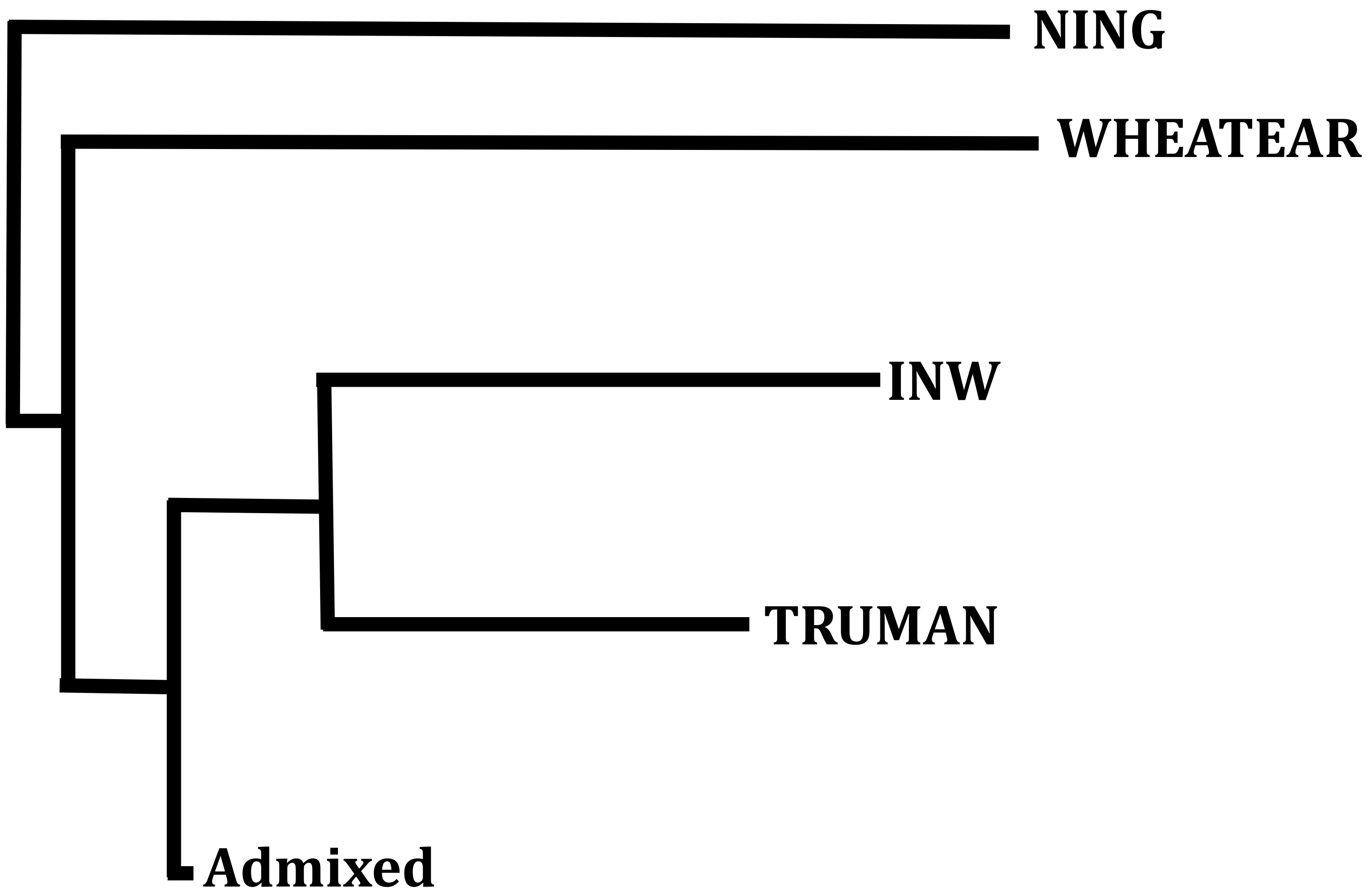
NING
WHEATEAR
INW
TRUMAN
Admixed
