# Supplementary material for: Identification of regions under selection and loci controlling agronomic traits in a soft red winter wheat population
Source: Plant Genome. 2020 Jun 29;13(2):e20031. doi: 10.1002/tpg2.20031 (PMC12807307; doi:10.1002/tpg2.20031)
Supplement: Supplementary file 2 [file TPG2-13-e20031-s001.docx]

**Supplemental Table TS1.** The table shows the LD decay distances for individual chromosomes. The chromosomes 2B and 7D with unusually high LD decay rates are underlined.

| Chromosome | Half decay distance (Mbp) |
| --- | --- |
| 1A | 2.64 |
| 1B | 6.10 |
| 1D | 4.97 |
| 2A | 3.01 |
| 2B | 763 |
| 2D | 12.93 |
| 3A | 1.93 |
| 3B | 3.15 |
| 3D | 1.88 |
| 4A | 2.77 |
| 4B | 5.42 |
| 4D | 0.64 |
| 5A | 4.69 |
| 5B | 5.37 |
| 5D | 1.68 |
| 6A | 2.73 |
| 6B | 3.82 |
| 6D | 0.77 |
| 7A | 1.69 |
| 7B | 3.05 |
| 7D | 94.32 |

**Supplemental Table TS2.** Summary of descriptive statistics and heritability of the six agronomic traits.

| Traits | Units | Mean | SD | Min | Max | *H^2^* | *H^2^_C_* |
| --- | --- | --- | --- | --- | --- | --- | --- |
| Days to heading | Julian days | 140.1 | 1.8 | 131.7 | 147.5 | 0.74 | 0.69 |
| Plant height | Cm | 78.5 | 7.8 | 55.4 | 124.7 | 0.84 | 0.78 |
| Test weight | Kg m^-3^ | 931.9 | 112.7 | 463.5 | 1282 | 0.49 | 0.41 |
| Grain yield | Ton ha^-1^ | 4.4 | 1.1 | 1 | 9 | 0.54 | 0.49 |
| #Spikes per unit area | Spikes m^-2^ | 710.7 | 121.4 | 337.8 | 1081.3 | 0.52 | 0.39 |
| #Kernels per unit area | Kernels m^-2^ | 15019.4 | 4530.1 | 2497.6 | 32378.6 | 0.4 | 0.27 |

*H^2^*: broad sense heritability estimated on entry mean.
